# Travelling in time with networks: Revealing present day hybridization versus ancestral polymorphism between two species of brown algae, *Fucus vesiculosus *and *F. spiralis*

**DOI:** 10.1186/1471-2148-11-33

**Published:** 2011-01-31

**Authors:** Yann Moalic, Sophie Arnaud-Haond, Cécile Perrin, Gareth A Pearson, Ester A Serrao

**Affiliations:** 1 IFREMER, Institut Français de Recherche pour l'Exploitation de la Mer, centre de Brest, BP70, 29280 Plouzané, France; 2 CCMAR, CIMAR, University of Algarve, Gambelas, 8005-139, Faro, Portugal

## Abstract

**Background:**

Hybridization or divergence between sympatric sister species provides a natural laboratory to study speciation processes. The shared polymorphism in sister species may either be ancestral or derive from hybridization, and the accuracy of analytic methods used thus far to derive convincing evidence for the occurrence of present day hybridization is largely debated.

**Results:**

Here we propose the application of network analysis to test for the occurrence of present day hybridization between the two species of brown algae *Fucus spiralis *and *F. vesiculosus*. Individual-centered networks were analyzed on the basis of microsatellite genotypes from North Africa to the Pacific American coast, through the North Atlantic. Two genetic distances integrating different time steps were used, the Rozenfeld (RD; based on alleles divergence) and the Shared Allele (SAD; based on alleles identity) distances. A diagnostic level of genotype divergence and clustering of individuals from each species was obtained through RD while screening for exchanges through putative hybridization was facilitated using SAD. Intermediate individuals linking both clusters on the RD network were those sampled at the limits of the sympatric zone in Northwest Iberia.

**Conclusion:**

These results suggesting rare hybridization were confirmed by simulation of hybrids and F2 with directed backcrosses. Comparison with the Bayesian method STRUCTURE confirmed the usefulness of both approaches and emphasized the reliability of network analysis to unravel and study hybridization

## Background

Speciation is a central process in evolution, but its complexity and duration render it difficult or impossible to observe and study as a whole. Studies dealing with the functional and genetic divergence between taxa, particularly when reproductive isolation is incomplete [1], allow the distinction and analysis of the various stages of this process. The relative influence of the different mechanisms involved in the initiation and maintenance of divergence can then be inferred. Understanding the complexity of evolutionary and ecological mechanisms leading to reproductive isolation and speciation through integration of *in situ *observations, theoretical models and molecular analysis of genomes, is today one of the major challenges in evolutionary ecology [2-6]. Important efforts towards modeling the processes acting in hybrid zones and understanding the maintenance of divergence have focused on the balance between dispersal and hybrid depression [7]. In these studies, incompatibility resulting from the allopatric divergence between two genomes is considered as a predominant factor causing hybrid depression. During the last decades, the development of the use of molecular markers in a population genetics framework made possible the testing and improvement of these theoretical models in hybrid zones. This screening of genome divergence and incompatibility also allowed testing the schematic models describing speciation as the result of processes spanning from pure vicariance (allopatry) to differential level of gene flow (sympatric speciation, [8-11]).

From a statistical point of view however, analyses at both the scales of populations and genes are still limited by the panel of tools available. At the population level, the analysis of molecular data is limited by the fact that most mathematical models underlying classical population genetics analysis have been developed in an intra-specific framework, and some underlying hypotheses such as random mating or Hardy Weinberg equilibrium do not necessarily stand in real natural populations and are out of scope in hybrid zones. Moreover, the detection and screening of hybrids requires an analysis of the populations and hybrid zones at the individual level whereas most summary statistics deliver estimates at the population level.

Two families of analyses have been proposed recently in order to partially release the underlying assumptions of most classical population genetics data analyses, and take better advantage of the information contained in a given dataset. The first family is built around the coalescent theory and is mostly based on Bayesian computation methods of analysis. It allows individual-centered analyses of genotype clustering [12-16]. The second family is built around the network theory and proposes an exploratory approach centered on the population or individual (also called agents), illustrating the relationship among those agents and inferring their respective roles and importance in the studied system [17-20].

Methods based on the coalescent theory trace the ancestral genealogy of a sample rather than modeling changes of gene frequencies in the population as a whole. These methods have recently been explored in order to improve mathematical models and take advantage of all the information contained in the data [12,21,22]. They have proven useful in studying hybrid zones [23] although they remain complex and time-consuming. On the other hand, this combination between high load of information not being optimally exploited by the summary statistics, and statistical tools available requiring heavy underlying hypotheses, suggests the use of another possible family of methods, coming from science of complex systems based on network theory [24,25]. Network tools have indeed been developed to take the best advantage of the information contained in a complex data set, while minimizing the assumptions required for the analysis and interpretation of the complex system behavior [26].

A first step towards the use of the network theory to unravel gene flow has recently been proposed for inter-population analyses by Dyer and Nason [17]. They applied the graph theory developed by Erdös and Renyi [1960, in 27] to describe the complex topology resulting from both history and contemporary genetic interactions among populations of a widely distributed species of cactus. This was further improved to extract from the graph topology hints as the dynamics of information (here gene) flow through the system at the inter-individual [19] and inter-population levels[20]. Compared to classical methods [28], these first steps proved useful in illustrating the genetic relationship, and identifying clusters of individuals as well as agents acting as preferential links or sources in the metapopulation system of a threatened seagrass.

In the present study, we explored the usefulness of network analysis to study hybrids identified as agents linking two differentiated clusters of individuals, or gene pools. We tested network analysis in the hybrid zone between our two model species, the two sister species of brown seaweeds *Fucus vesiculosus (F_ves) *and *F. spiralis (F_spi). *Both species are ecologically successful and widely distributed (North Atlantic, Channel and North Sea shores). They are characteristic of respectively upper and mid-shore zones on rocky shores. Although distinct genetic entities have been identified within the species *F. spiralis *[*F. spiralis*-High and *F. spiralis*-Low, [29]] these are nevertheless still one single monophyletic entity, here designated as *F. spiralis*, distinct from its sister *F. vesiculosus *[30]. These two sister taxa are a good model system because, despite displaying diagnostic reproductive system (*F_ves *is dioecious whereas *F_spi *is hermaphroditic), they may hybridize when encountered in sympatry [31-34], resulting in individuals with intermediate genotypes whose fitness in terms of reproductive investment is not significantly reduced [35]. Whether the occurrence of hybridization is the result of an ongoing and incomplete speciation process or of a secondary contact with ongoing re-homogenization of the two entities is still unclear and a matter of debate [36,37]. In order to test for the hypothesis of ongoing speciation (i.e., ancestral shared polymorphism) versus present day hybridization due to secondary contact (introgression), we studied the genetic relationships among samples collected both in sympatric and allopatric regions of *F_ves *and *F_spi *ranges (see FigureA1 in additional file 1). The global pattern of genetic relationships among individuals was illustrated by networks built with two different metrics integrating different genetic information in term of time and divergence history: the Rozenfeld distance (RD) [19] and the Shared Allele distance (SAD) [38]. RD, based on loci, helps to resolve ancestral polymorphism through allele length impinged by slow evolutionary processes, while SAD, based on shared alleles, helps to understand recent gene flow characterized by direct allelic exchange free from slow evolutionary process. We aimed at comparing the performance of network and Bayesian coalescent analysis (STRUCTURE, [16]) to test whether the shared polymorphism and the absence of diagnostic locus was mainly due to retained ancestral polymorphism, or to present day hybridization in sympatric zone. In the first case, we expected shared polymorphism at neutral microsatellites to be distributed evenly across the species distribution range, whereas in the other case a higher proximity among species may be expected in sympatric zones.

## Results

### Networks of individuals F. spiralis and F. vesiculosus

**Figure 1:**
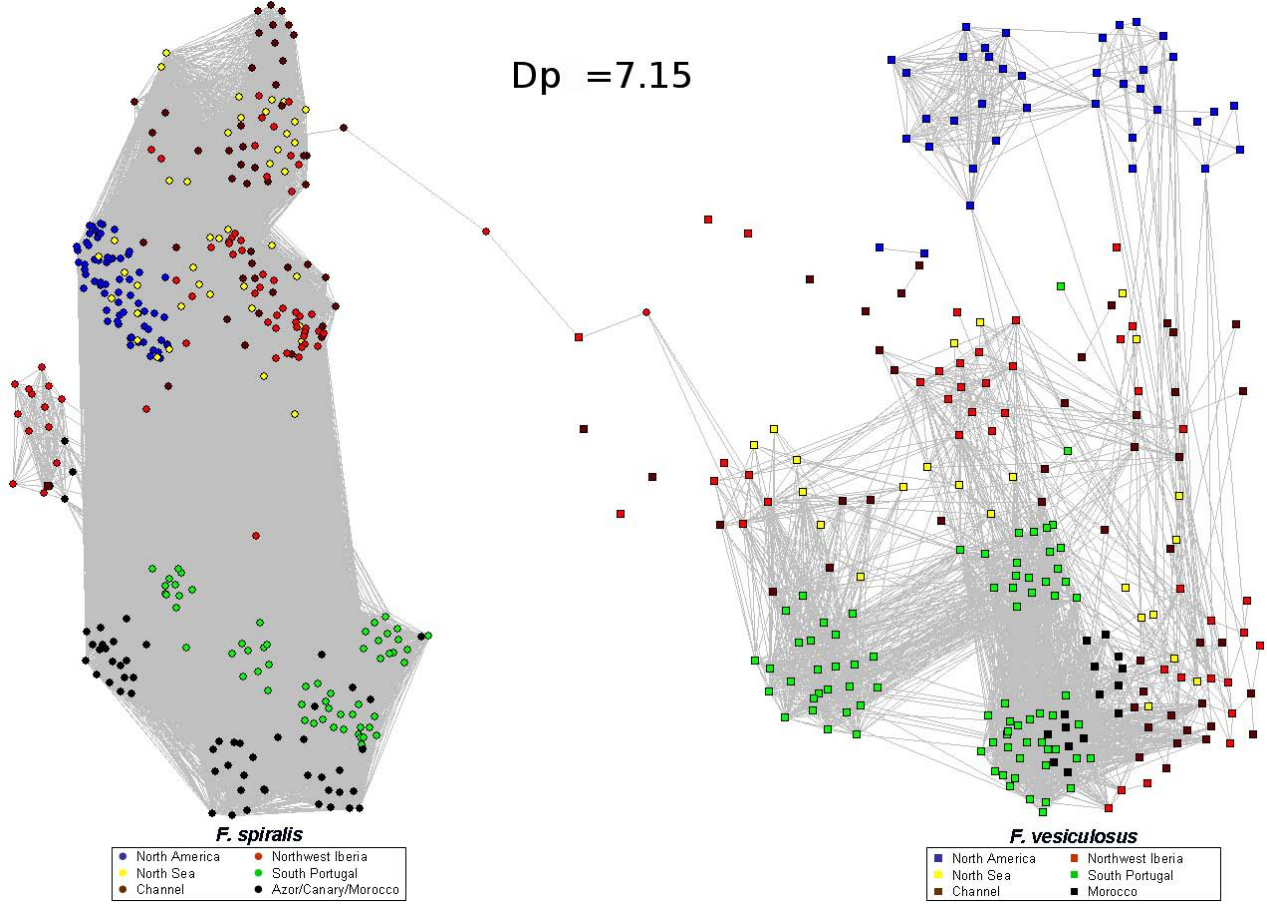
**Network topology of *F. spiralis *and *F. vesiculosus *individuals with the Rozenfeld Distance (RD)**. Only links with value smaller than or equal to the percolation distance Dp = 7.15 are present. Nodes representing individuals are circles for *F. spiralis *and squares for *F. vesiculosus*. Colors correspond to geographical regions. One can identify 2 clusters, one for *F. spiralis *individuals and the other for *F. vesiculosus*.

Networks were analyzed at the percolation threshold (see methods). This approach is based on the analysis of the network keeping only essential links illustrating the minimum genetic distance necessary to maintain connectivity across most components of the system. The network based on the Rozenfeld distance (RD; Figure 1) reveals diagnostic RD distances among both species. At the percolation threshold distance (Dp = 7.15), genotypes corresponding to sampled *F_spi *(left) versus *F_ves *(right) clearly segregate into two sub-clusters linked through a succession of 4 nodes (Figure 1). Below this percolation threshold, the network loses its integrity and the species-specific sub-clusters split into distinct clusters (see Figure A2 in additional file 2), each exclusively composed of either *F_spi *or of *F_ves *individuals. The ensuing clusters of individuals present a hierarchical structure supported by a significant average clustering coefficient: < CC > = 0.72 higher than the one expected after randomly rewiring the links (< CC_o _> = 0.11 with σ_o _= 0.1, after 10,000 random simulations). Thus illustrating the modularity of the network composed of two clusters of species, with greater internal interconnection than would be expected by chance.

**Figure 2:**
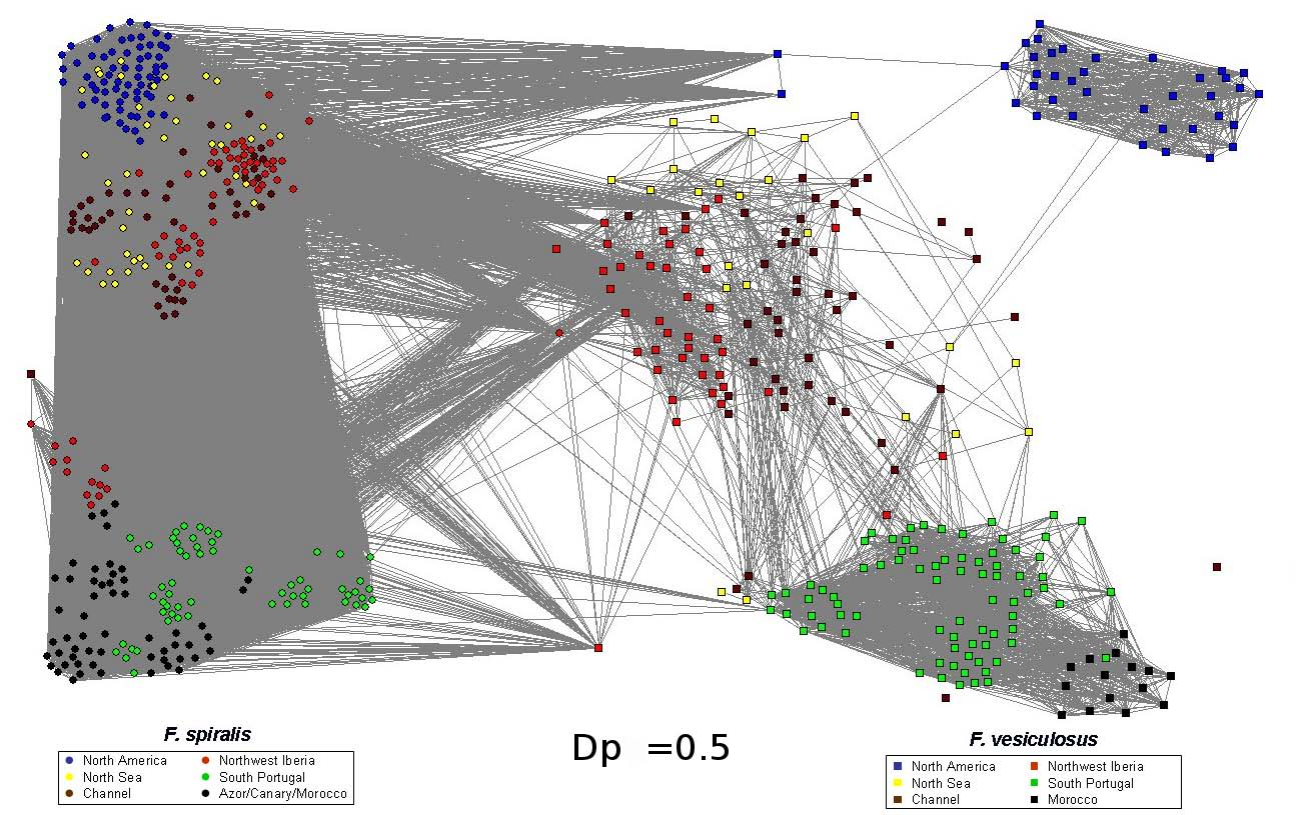
**Network topology of *F. spiralis *and *F. vesiculosus *individuals with the Shared Alleles Distance (SAD)**. Only links with value smaller than or equal to the percolation distance Dp = 0.5 are present. Nodes representing individuals are circles for *F. spiralis *and squares for *F. vesiculosus*. Colors correspond to geographical regions.

The network based on Shared Alleles distance (SAD), only influenced by allele frequencies and not by their divergence, does not discriminate the species as clearly as the RD did (Figure 2). Its different topology reveals hierarchical clustering reflected already on the percolation profile (see methods and Figure A3 in additional file 3) where the single peak with RD is replaced by three peaks with the SAD. At the percolation threshold (Dp = 0.5), the segregation of the sister species is not as clear as with RD. A large number of pathways still connect the two species and the decrease of threshold does not lead to a clear separation of *F_spi *and *F_ves*. Indeed, the first disconnecting cluster corresponds to the Northern American *F_ves *(thr = 0.39, see Figure A4 A in additional file 4), followed by the cluster of *F_ves *from South Portugal and Morocco (thr = 0.33, see Figure A4 B in additional file 4) while the last cluster of *F_ves *from the sympatric zone of Northwest Iberia (i.e., Northwestern Spain and Northern Portugal) remains linked to the complete cluster of *F_spi*. These observations are consistent with a significant average clustering coefficient value inferior with SAD than RD: < CC > = 0.46 (< CC_o _> = 0.17 with σ_o _= 0.1, after 10,000 random simulations).

The individuals of *F_spi *that are sharing the higher number of links with individuals *F_ves *were also coming from Northwest Iberia. Despite the global differences denoted for our networks, this last observation is common to both the SAD and RD network topologies. Indeed, both highlight individuals of *F_spi *and of *F_ves *from Northwest Iberia relaying gene flow among their two clusters. The combination of our two network analyses with different distances indicates hybridization in our dataset at the Northwest Iberia area.

### Hybridization assessment through network analysis

As the network topology built with SAD was not discriminative for the 2 species, a method was developed to derive expectations of SAD network topology under the hypothesis of hybridization (see Hybridization simulation methods in Additional file 5). Indeed, at the threshold value of 0.39, on the network topology (see Figure A4 A in additional file 4), some natural individuals of *F_spi *and *F_ves *of the sympatric zone of North Iberia are making the link between the two species. These 17 intermediate individuals were considered as putative hybrids. The threshold value of 0.39 was selected because of the background noise at 0.5 illustrated by direct links between individuals of *F_spi *and *F_ves *coming from different geographic area. Hybridization simulations were thus conducted under 4 different conditions (Table 1) and network analysis was employed to compare the behavior of these hybrids when integrated into the initial natural dataset (hereafter called hybrids datasets).

**Table 1:** Hybrids datasets used in this study

	number of simulated hybrids	number of natural individuals	total number of individuals
Hybrids F1	17	555	572
BC_*F_spi*	17	555	572
BC_*F_ves*	17	555	572
BC_*F_spi_F_ves*	17 (9+8)	555	572

**Figure 3:**
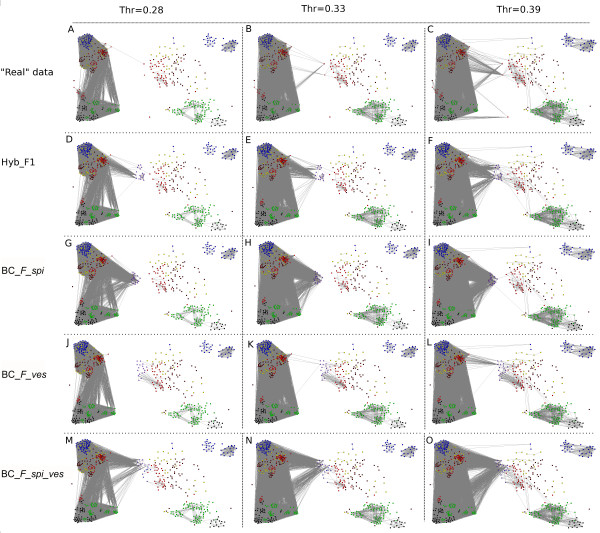
**Network topology of *F. spiralis *and *F. vesiculosus *individuals + simulated hybrids with the SAD**. Only links with value smaller than or equal to the threshold values are present. Nodes representing individuals are circles for *F. spiralis *and squares *for F. vesiculosus*. Nodes representing hybrids are triangles. Colors correspond to geographical regions.

The topologies of derived networks were compared across the 4 hybrids datasets at decreasing thresholds, 0.39, 0.33 and 0.28 (Figure 3) allowing networks to pass from a connected state to a disconnected one preventing gene flow between the two species. At the threshold of 0.39, *F_spi *are connected to *F_ves *through synthetic hybrids for all datasets. Nevertheless, differences appear during the decrease of the threshold highlighting the closer behavior of BC_*F_ves *and BC_*F_spi_F_ves *hybrids datasets to the initial natural dataset than hybrids F1 and BC_*F_spi*.

**Figure 4:**
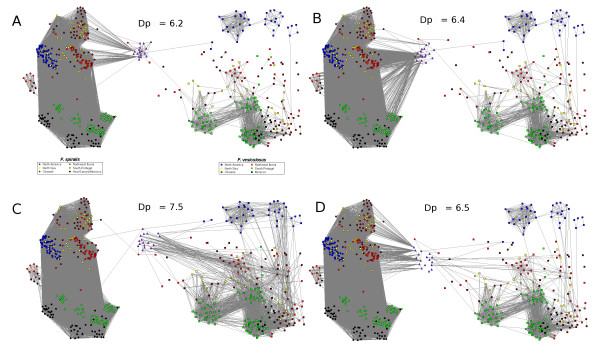
**Network topology of *F. spiralis *and *F. vesiculosus *individuals + simulated hybrids with the RD**. Only links with value smaller than or equal to the percolation thresholds are present. Nodes representing individuals are circles for *F. spiralis *and square for *F. vesiculosus*. Nodes representing hybrids are triangles. (A) Hybrids F1 (B) hybrids BC_*F_spi*, (C) hybrids BC_*F_ves*, (D) hybrids BC_*F_spi_F_ves*, blue triangle are *F_ves *hybrids and purple triangles are *F_spi*. Colors correspond to geographical regions.

The comparison of network topologies with RD and SAD helped to test for the hybridization scenario best fitting the natural dataset network topology. As revealed by the percolation curves (see Figure A5 in additional file 6), the percolation thresholds shift globally to lower values except for the BC_*F_ves *dataset (Dp = 7.5 vs. Dp = 7.15 for the natural dataset). As for the natural dataset, at the percolation threshold, hybrids datasets have only one pathway making the connection between each of the two clusters of species (Figure 4). Nevertheless, the network topologies, according to the percolation threshold, reveal an intermediary state between BC_*F_ves *and BC_*F_spi_F_ves *datasets where the natural dataset seems to fit in.

### Hybridization assessment through a Bayesian clustering approach

**Figure 5:**
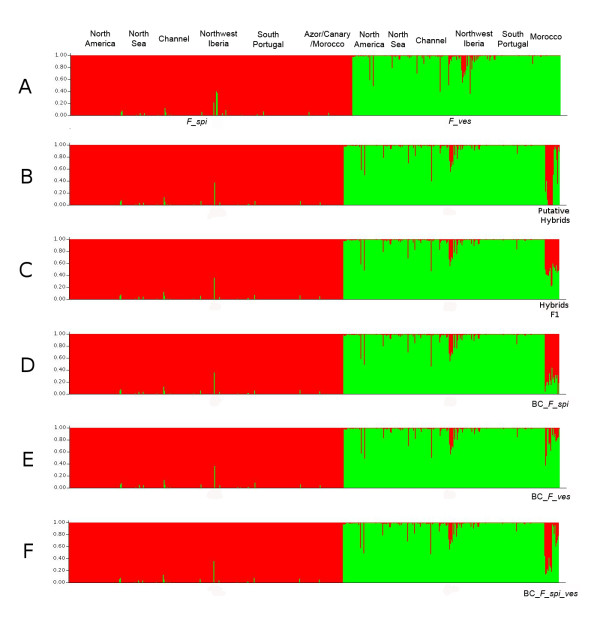
**Microsatellite detection of admixture (introgression) using the program STRUCTURE (Pritchard et al., 2000)**. Each individual is represented in the figure by a vertical bar and its colors indicate the proportional membership in each of k = 2 clusters, thereby providing a quantitative illustration of introgression. Red represents *F. spiralis*, green *F. vesiculosus*.

In order to highlight the potential differences existing between the two methods and to depict their advantages and disadvantages in hybridization assessment, we compared the networks with the software STRUCTURE [16], that assigns genotypes proportionally to clusters defined based on minimizing linkage and Hardy-Weinberg disequilibria. The results obtained with STRUCTURE for two clusters (k = 2) are coherent with mainly two categories of individuals in agreement with the morphological determination of the natural dataset of all individuals into the two species (Figure 5A). Nevertheless, 21 intermediate individual genotypes (more than 10% of ancestry coming from one of the 2 species) are counted: 3 *F_spi *and 18 *F_ves*. It appears that only few of them (n = 5, 2 *F_spi *and 3 *F_ves*; n_s _= 1+1) are among the 17 putative hybrids detected with the SAD network and relocated at the end of the dataset (Figure 5B). It is also noticeable that although most of the individuals (n = 13, n_s _= 3) considered as admixed in STRUCTURE are in the sympatric zone of Northwest Iberia, some (n = 8, n_s _= 4) do not have a clearly intermediate position on the SAD network, with even one coming from an allopatric area (Southwest Portugal).

When analyzing the datasets where putative natural hybrids were replaced by synthetic hybrids, admixture is mainly recovered. As with network analysis, all individuals of Hybrids F1 are clearly recognized as such by STRUCTURE (Figure 5C). This result is coherent with the network topology, as for backcrosses BC_*F_spi*, BC_*F_ves *and BC_*F_spi_F_ves*, 15/17, 11/17 and 14/17 (9+5) individuals are detected, respectively (Figure 5D, E and 5F). Nevertheless, it should be noticed that individuals BC_*F_ves *are less detected by STRUCTURE as admixed than BC_*F_spi *(9 vs. 2 on the totality of backcrosses). This result is different from the network analyses because individuals BC_*F_spi *tend to be closer to natural individuals of *F_spi *than BC_*F_ves *to natural individuals of *F_ves*.

## Discussion

In this study, we developed a novel application of network analysis to the study of hybridization phenomena between sister species. Networks were constructed based on distinct genetic distances (RD and SAD), differently sensitive to time since divergence (respectively based on allele lengths or on shared alleles), allowing the comparison of the divergence degree at different time-scales.

The first important picture obtained on network built with Rozenfeld Distance (RD) is the straightforward recognition of two well-defined clusters of *F. vesiculosus *and *F. spiralis *(Figure 1). This confirms that reproductive isolation and genetic divergence is rather advanced between those two species, despite a significant amount of shared polymorphism. Thus this multi-locus phylogenetic distance may be used to assign individuals to their species of origin. This result also supports the existence of phylogenetic information from microsatellite loci [39] which is accurately delivered by RD.

The second important result is the occurrence of clusters of individuals exhibiting an intermediate position between both species and maintaining a connection between some *F. vesiculosus *populations and the *F. spiralis *cluster as revealed on the SAD network (Figures 2 and 3). This intermediate cluster is formed by tens of individuals, among which those pointed out as intermediate with the RD. In case of the shared polymorphism being mostly ancestral, one may expect to observe some connections anywhere in the distribution range including in allopatric zones, but these *F. vesiculosus *individuals of intermediate genotypes are only detected in the Northwest Iberia, located specifically at the edge of the locations where both species occur in sympatry. Interestingly, intermediate individuals, although much less numerous, also emerge in two other sympatric areas: Northeastern America and the English Channel, whereas no such individuals are observed in any of the allopatric zones, further supporting the hybridization hypothesis. The SAD distance integrates more recent history, therefore giving it more weight than the RD, which takes into account phylogenetic divergence [19,38]. It is therefore likely to better reflect present day exchange of genes between the two species. Those intermediate individuals that did cluster with the main *F. vesiculosus *cluster with RD, with SAD do now connect closer to *F. spiralis *than to the other con-specifics. This is likely caused by these individuals sharing more alleles with *F. spiralis *than with the other *F. vesiculosus *(therefore the branching with SAD distance), but the alleles they do not share with *F. spiralis *exhibit a strong divergence with this last species and are typical from *F. vesiculosus *(therefore the clustering of those individuals with *F. vesiculosus *with RD). The occurrence of all those individuals in the sympatric zone supports these intermediate genotypes as the product of present day hybridization between anciently diverged lineages/species.

The four additional networks built including simulated synthetic hybrids from F1, as well as backcrosses with each of the mother species (Figure 3), confirm this scenario. Indeed, the synthetic hybrids are present at the interface of the two sister-species, although they tend to be more isolated than most of the natural putative hybrids. This isolation seems due to the F1 nature of synthetic hybrids, truly half *F. spiralis*/half *F. vesiculosus*, while naturally occurring hybrids may be the product of backcrosses. When the backcrosses are plugged into the network, all possible backcrosses do not lead to a convergence towards the initial topology based on real data only. The admixture of (F1 X *F. spiralis*) and (F1 X *F. vesiculosus*) in the same dataset show the most similar network topology to the original one, while backcrosses resulting from (F1 X *F. spiralis*) or (F1 X *F. vesiculosus*) plugged alone tend to cluster closer to their species of origin without fitting exactly the same position as the natural intermediates.

The use of network here provides specific advantages, mainly the ability to identify agents (nodes) connecting identified clusters through genetic distance (links). Hybridization indeed usually results in a reticulate tree that cannot be built with classical phylogenetic methods but can be easily grasped on a network. Moreover, the possibility to follow the evolution of network topology through a gradient of genetic distance threshold helps to visualize and understand the attachment preferences to one or the other of the species clusters.

The use of STRUCTURE also illustrates the clear separation between the two species and allows the identification of admixed individuals, although not systematically the same as the intermediate agents appearing on the networks. In order to test for the accuracy of each method in those doubtful cases, the discrepancies between results obtained with both methods can be considered in perspective with geographical locations and ecological conditions in which intermediate agents (network) do not appear as admixed individuals (STRUCTURE), or the other way round. Interestingly, intermediate agents unidentified by STRUCTURE as admixed individuals correspond to samples collected at the edge of the zone where the two species occur in sympatry, Northwest Iberia. On the contrary, the admixed individuals detected by STRUCTURE that do not appear as intermediate agents were collected in the allopatric zone, thereby rendering enigmatic the origin of such admixture.

When analyzing the accuracy of both methods with synthetic hybrids and backcrosses, STRUCTURE shows high reliability in detecting F1 hybrids (100%), whereas only 71% of those are emphasized by an intermediate position on network. The detection of mixed individuals with STRUCTURE logically decreases to 92% for backcrosses with *F. spiralis *and to 64% for backcrosses with *F. vesiculosus*. This difference between the two species can be explained by the lower genetic diversity of *F_spi*, likely due to its reproductive mode, as illustrated by their GDS (see Figure A6 in additional file 7), resulting in an easier detection of the insertion of new alleles in the genetic pool. On the opposite, network analysis, by relying on shared links, shows backcrosses with *F_spi *having a stronger assimilation to its species of origin than backcrosses with *F_ves*. The integration of backcross events into the species gene pool seems to be rapid and renders them hard to identify. As a synthesis, both approaches appear as complementary, as STRUCTURE may perform slightly better in the systematic detection of admixed individuals, an advantage however balanced by a lower amount of misleading detection of hybrids (i.e. type I errors) obtained with network analysis. Besides, network analysis allows further screening of links and connections among geographic areas to describe patterns of spatial connectivity.

At the within species scale, finally, network analysis revealed two main genetically distinct clusters across the distributional range of both sister species *F. spiralis *and *F. vesiculosus*, (a) a Southern cluster in South Portugal, Morocco, Azores and Canary Islands, and (b) and a Northern cluster in North America, North Sea and Channel (see Figures A2 B and A4 B in additional files 2 and 4). These two regions are common to both species indicating a similar evolutionary history. Indeed, oscillations of climate during the past thousands of years have caused repeated geographic distributional shifts and extinction/recolonization events often experienced by many marine taxa. During the Last Glacial Maximum (LGM, 23-18 ka) in Europe, permafrost extended south at 47° N [40], leaving temperate species to shift their distribution to potential refugia. Marine species, including intertidal taxa, are also thought to have been displaced to small refugia in the North around the British Isles, Norway or in the Brittany region and more Southerly from the Iberian Peninsula to Mauritania [41]. As the ice melted, species ranges were able to expand back to previous latitudes [42,43]. On the North American Coast, the LGM may have covered by ice the complete hard substrate available there [44] and have caused the extinction of rocky shore species in this region. North American rocky shores are thought to have later been recolonized by European populations [45]. For species in the genus *Fucus*, glacial refugia have been inferred along the Brittany area for *F. serratus *[45] or along Northwest Iberia for *F. ceranoides *[46] and *F. vesiculosus *along the American coast shows a genetic signature of a recent recolonization from Europe [47]. In *F. spiralis *and *F. vesiculosus*, clustering on our networks is consistent with the presence of two refuge areas in Europe, possibly a Northern refuge (possibly along the Brittany or North Iberian region) and a southern one (possibly located along the southern Iberian Peninsula and/or North Africa). These inferences are thus consistent with a recent mitochondrial phylogeography of these two species for *F. spiralis *but are contradictory for *F. vesiculosus*, as the mitochondrial genome of *F. vesiculosus *supports a single refugial zone [32]. Organelle genomes, with smaller effective population size than nuclear ones, are however more prone to being highly affected by introgressive sweeps, and indeed introgression and massive expansion of *F. vesiculosus *organelles into other *Fucus *species has been documented [32,46]. Isolation into distinct glacial refugia would have been followed by a post-glacial expansion during which the Northern and Southern populations might have converged in a contact region along Northwest Iberia (Northern Portugal/Northwestern Spain) where despite hybridization genetic differentiation is still maintained nowadays (see Figures A2 B and A4 B in additional files 2 and 4) and which now also extends into the Brittany region [29,30,32], although in regions not included in our sampling. In the southern region, *F. spiralis *occurs only on the open coast whereas *F. vesiculosus *is present only in isolated sheltered areas such as estuaries and coastal lagoons [48]. Consequently, along the broad areas of sympatry of their Northern distribution, the two sister species can hybridize [33,34,49], whereas in Southern Europe, their distribution is allopatric and hybridization is highly unlikely due to the limited dispersal capability of their gametes [50].

A more complex pattern appears on the networks in Northwest Iberia area (Figure 2). The Northwest Iberian close connection to the Northern cluster (North Sea and Channel) indicates a recent secondary expansion either from North Iberia into the Northern region or *vice versa*, having kept restricted gene flow with the southern clusters.

Another interesting point is the fact that hybrids are mostly localized in the Southern limit of the sympatric zone, where it contacts the allopatric zone ranging from Southern Portugal-Northwestern Africa and Atlantic islands. Hybridization may be favored by unknown factors in this area, such as the rate of hybrid fertility that may change between geographical locations, possibly being higher in Northwest Iberia area as suggest by Billard et al. [49]. Also, reinforcement may be weak due to the recent gene flow from an allopatric zone where it would be lacking, whereas in the middle of the sympatric zone such mechanism would already have developed to maintain species integrity. This is further supported by the peculiar position and clustering of the individuals from the extreme edge of the sympatric zone in Mindelo, where more than half intermediate individuals were detected on the network, and which is the only sympatric population of *F. spiralis *clustering with the allopatric ones with Structure (k = 3; see Figure A7 in additional file 8). These results are in agreement with a recent multi-gene phylogeny of these species that reveals that in Northwest Iberia *F. spiralis *from southern origins become extremely introgressed [30].

## Conclusions

The results of the present study suggest three main conclusions: (i) The accuracy of network analysis to unravel hybridization phenomena. The detection of hybrids and introgression is possible and reliable through network analysis, although not systematic as some remain apparently undetected in any analysis attempted, either networks or STRUCTURE. (ii) The putative hybrids detected in the SAD network seem more similar to backcrosses (indifferently with one or the other of the parental species) than to F1 Hybrids. This may be interpreted as a seldom occurrence of first generation hybrids, with a dilution effect on the mosaic genomes produced after backcross events. (iii) The biogeography of both *Fucus *species addressed through the examination of networks supports the existence of Northern and Southern glacial refuges where both species differentiated in two clusters that came back into contact in Northwest Iberia during post-glacial range changes. Interestingly, this area corresponds to the boundary between the allopatric/sympatric zones, where most putative hybrids were detected, suggesting weaker reinforcement in this area.

## Methods

### Dataset used in this study

A total of 572 individuals of *F_spi *and *F_ves *(respectively 329 and 243) were collected throughout the North Hemisphere (see FigureA1 and TableA1 for location distribution in additional files 1 and 9). The analysis covers their entire latitudinal range. A combination of nine microsatellite markers was selected for their amplification consistency and polymorphism in both species. The marker combination includes L20-58-78-38-94 [34], Fsp 1-2-4 [51] and F90 [33].

### Genetic distances used in this study

Two different genetic distances taking or not phylogenetic information into account were chosen to perform network analysis of our dataset.

First, we used the Goldstein distance for populations [52] modified for use between individuals, as originally presented by Rozenfeld et al. [19]. As the underlying data are based on multilocus microsatellite genotypes, an individual is characterized by a series of pairs of microsatellite repetitions at k loci with k = 9.

More precisely, the genotype of an individual, called A, is represented as

$$A=(a_{1},A_{1})(a_{2},A_{2})...(a_{k},A_{k}),$$

where a_i _and A_i _are the allele length (in number of nucleotides) in both chromosomes at locus i.

Given a second individual, B, with genotype

$$B=(b_{1},B_{1})(b_{i},B_{i})...(b_{k},B_{k}),$$

we define a dissimilarity degree between A and B at locus i as

$$d_{i}(A,B)=\min\left(\left|A_{i}-B_{i}\right|+\left|a_{i}-b_{i}\right|,\left|A_{i}-b_{i}\right|+\left|a_{i}-B_{i}\right|\right),$$

which provides a parsimonious (i.e. minimal) representation of the genetic distance, understood as the difference in allele length, between samples A and B.

Then we define the Rozenfeld distance (RD) among *Fucus *individuals by averaging the contributions from all loci

$$D(A,B)=\frac{1}{k}\sum_{i=1}^{k}d_{i}$$

The second genetic distance used is the Shared Allele distance (SAD). This genetic distance is based on the proportion of shared alleles [38]. For individual pairwise comparisons the proportion of shared alleles is estimated by:

$$P_{SA}=\frac{1}{2n_{u}}\sum_{u}S,$$

where the number of shared alleles S is summed over all loci u and n_u _is the number of loci.

Distance between individuals,

$$D_{SA}=1-P_{SA},$$

This individual measure can be used to look at population substructure. Bowcock et al. [53] constructed dendrograms based on this distance calculated from human microsatellite data. Using this technique, a correlation between genetic similarity and geographic location was noted. This distance measure has also proven very successful at placing unknown individuals into the correct subpopulation [54].

Finally, these distances help to resolve the relationship between individuals at different time-scale. RD, based on loci, helps to resolve individuals' origin at an older time, while SAD, based on shared alleles, helps to understand recent gene flow.

### Network analysis

Once we have calculated the matrices of genetic distances between individuals described above containing all the individuals of our dataset, we built networks by considering individuals as nodes and genetic distances between them as links.

As we aimed to both visualize hybridization phenomenon on the network and localize its occurrence geographically, we started to remove links in a decreasing order starting from the one with the highest value until the network collapses. Just before this state, the percolation distance (Dp) is reached [55] and small clusters of fields start to be released. This phenomenon can be interpreted as the time when the integrity of the gene flow is stopped all across the network. A standard methodology to calculate this value for finite system consists of calculating the average cluster size of the cluster excluding the largest one [55],

$$\langle S\rangle *=\frac{1}{N}\sum_{s\langle S\max}s²ns,$$

as a function of the last distance value removed, thr. N is the total number of nodes not included in the largest cluster and n_s _is the number of clusters containing s nodes. The resulting curves show a maximum followed by a strong decrease where the percolation threshold is positioned. Once this percolation threshold is identified, we analyzed the network topology and its characteristics at this point.

### Estimate the global and local properties of the Network

The different indexes of network theory used to describe and characterize our network are:

The connectivity degree k_i _of a given node i is the number of other nodes linked to it (i.e., the number of neighbor nodes).

The distribution P(k) gives the proportion of nodes in the network having degree k.

The number of links E_i _existing among the neighbours of node i. This quantity takes values between 0 and E_i_^(max) ^= k_i_(k_i_-1)/2, which is the case of a fully connected neighborhood.

The clustering coefficient C_i _of node i is defined as

$$Ci=\frac{Ei}{Ei^{\left(\max\right)}}=\frac{2Ei}{ki\left(ki-1\right)}.$$

It quantifies how close the node i and its neighbors are to being a clique (complete graph).

The clustering coefficient of the whole network < CC > is defined as the average of all individual clustering coefficients in the system.

The betweenness centrality [56] of node i, bc(i), counts the fraction of shortest paths between pairs of nodes that pass through node i. Let σ_st _denote the number of shortest paths connecting nodes s and t and σ_st_(i) the number of those passing through the node i. Then,

$$bc(i)=\sum_{s\neq t\neq i}\frac{\sigma st\left(i\right)}{\sigma st}.$$

### Genetic diversity spectrum

The genetic distance between pairs of individuals within all locations was calculated in order to plot the frequency distribution of all pairwise values. This distribution is referred as the genetic diversity spectrum (GDS) as defined by Rozenfeld et al. [19].

### Hybrids/backcrosses simulations

We generated hybrids by random simulation of individuals of which the genotype is half *F_ves*/half *F_spi*. To do so, we used a random generator PERL script that selects randomly natural individuals present in locations where hybridization was suspected according to the SD network topology (see results). Then, for each of the nine loci, alleles (one from *F_ves *and the other from *F_spi*) were randomly chosen to create hybrids of first generation (F1). Among the natural individuals that were chosen, we excluded the natural putative hybrids, detected at the interface of the clusters of individuals *F. spiralis *and *F. vesiculosus *(see Table A2 in additional file 10), in order to avoid backcross hybridization in the same set of simulations. The natural putative hybrids are the individuals *F. spiralis *and *F. vesiculosus *connected between them allowing the information flow between the clusters of the species inside the network.

For the backcrosses, we followed the same scheme but we changed one of the natural individual by a hybrid F1. For example, backcrosses *F. spiralis *are the fusion of natural *F. spiralis *individuals and the hybrids F1. The detailed methodology used to generate hybrids and insert them into the networks is available in additional file 5.

### Admixture proportions of all individuals

In order to test for the ability of the network analysis to detect hybrids, we compared its efficiency with the program STRUCTURE [16]. This method has been previously used in the literature to identify species and hybrids in these species of *Fucus*, using microsatellite data [29,34]. STRUCTURE was run on both raw data and on data including synthetic hybrids for two clusters (k = 2) assuming admixture and independent allele frequencies between *F_spi *and *F_ves*. Analyses were performed using a burn-in period of 5,000 followed by 10,000 Markov Chain Monte Carlo repetitions. Individuals were considered as intermediate genotypes when they have more than 10% of ancestry coming from one of the 2 species. The confidence interval was computed ('print credible region' parameter) and intermediate genotypes labeled as 'significantly admixed' when the confidence interval was strictly included between 0.1 and 0.9. Results are detailed mentioning n as the number of admixed individuals (admixture proportion included between 0.1 and 0.9) and n_s _the number of those admixed individuals included in the 90% confidence interval.

## Authors' contributions

The network approach to the hybridization/polymorphism research question was conceived by SAH and EAS, the question of hybridization/polymorphism in allopatry versus sympatry was conceived by EAS, CP and GAP. EAS, GAP and CP performed some of the sampling. CP and SAH performed some of the genotyping. YM performed all the statistical analyses. YM, SAH and EAS interpreted the results. YM, SAH, CP and EAS wrote the paper. All authors have read and approved the final manuscript.

## Supplementary Material

Additional file 1 **Figure A1: Map of the study locations.** Colors correspond to geographical regions as on network figures. Blue is America, brown is North Sea, yellow is the Channel, red is Northwest Iberia, green is South Portugal and black is Azores/Canary/Morocco. East America, North Sea, the Channel and Northwest Iberia are sympatric sites while the others are allopatric. See Table A1 for precise locations. Click here for file

Additional file 2 **Figure A2: Network topology of *F. spiralis *and *F. vesiculosus *individuals with the Rozenfeld distance under the percolation threshold.** At the threshold values of 7.11 (A) and 4 (B). Nodes representing individuals are circles for *F. spiralis *and squares for *F. vesiculosus*. Colors correspond to geographical regions (see Figure A1). One can see at D = 7.11, the network is composed of two giant clusters corresponding respectively to the two species. At D = 4, the clusters of *F. spiralis *is still entirety at the exception of a little cluster of North Portugal. Click here for file

Additional file 3 **Figure A3: The average cluster size excluding the largest one, as a function of the imposed genetic threshold obtained with the natural individuals.** (A) calculated with the Rozenfeld distance, (B) calculated with the Shared alleles distance. The arrows indicate the percolation threshold (Dp) on the curves. Click here for file

Additional file 4 **Figure A4: Network topology of *F. spiralis *and *F. vesiculosus *individuals with the Shared alleles distance under the percolation threshold.** At the threshold values of 0.39 (A) and 0.33 (B). Nodes representing individuals are circles for *F. spiralis *and squares for *F. vesiculosus*. Colors correspond to geographical regions (see Figure A1). Click here for file

Additional file 5 **Hybridization simulation methods**. This file includes the description of the method used to randomly generate individual hybrids. Click here for file

Additional file 6 **Figure A5: The average cluster size excluding the largest one, as a function of the imposed genetic threshold calculated with the Rozenfeld distance.** (A) is the curve obtained with the simulated hybrids *spiralis/vesiculosus*, (B) with back-crosses hybrids/*spiralis*, (C) back-crosses hybrids/*vesiculosus *and (D) with back-crosses hybrids/*spiralis_vesiculosus*. The arrows indicate the percolation threshold (Dp) on the curves. Click here for file

Additional file 7 Figure A6. The genetic diversity spectrum (GSD) for *F. spiralis *(A) and *F. vesiculosus *(B) based on the Rozenfeld distance. Click here for file

Additional file 8 **Figure A7: Microsatellite detection of admixture (introgression) using the program STRUCTURE (Pritchard et al., 2000).** Each individual is represented in the figure by a vertical bar and its colors indicate the proportional membership in each of k = 3 clusters, thereby providing a quantitative illustration of introgression. Click here for file

Additional file 9 Table A1: Global characteristics of the dataset used in this study. Click here for file

Additional file 10 Table A2: Natural putative hybrids highlighted by the SD network. Click here for file
