# Supplementary figures and images for: Travelling in time with networks: Revealing present day hybridization versus ancestral polymorphism between two species of brown algae, Fucus vesiculosus and F. spiralis
Source: BMC Evol Biol. 2011 Jan 31;11:33. doi: 10.1186/1471-2148-11-33 (PMC3040711; doi:10.1186/1471-2148-11-33)

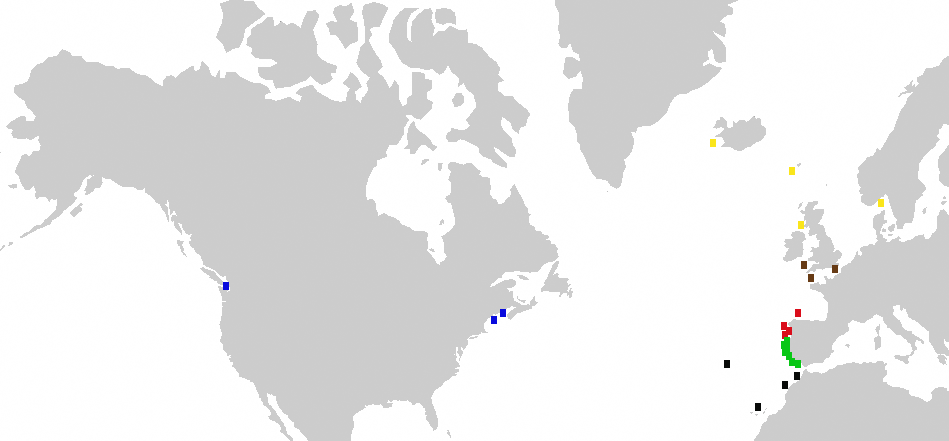

Supplement: Additional file 1 — Figure A1: Map of the study locations. Colors correspond to geographical regions as on network figures. Blue is America, brown is North Sea, yellow is the Channel, red is Northwest Iberia, green is South Portugal and black is Azores/Canary/Morocco. East America, North Sea, the Channel and Northwest Iberia are sympatric sites while the others are allopatric. See Table A1 for precise locations. [file 1471-2148-11-33-S1.TIFF]

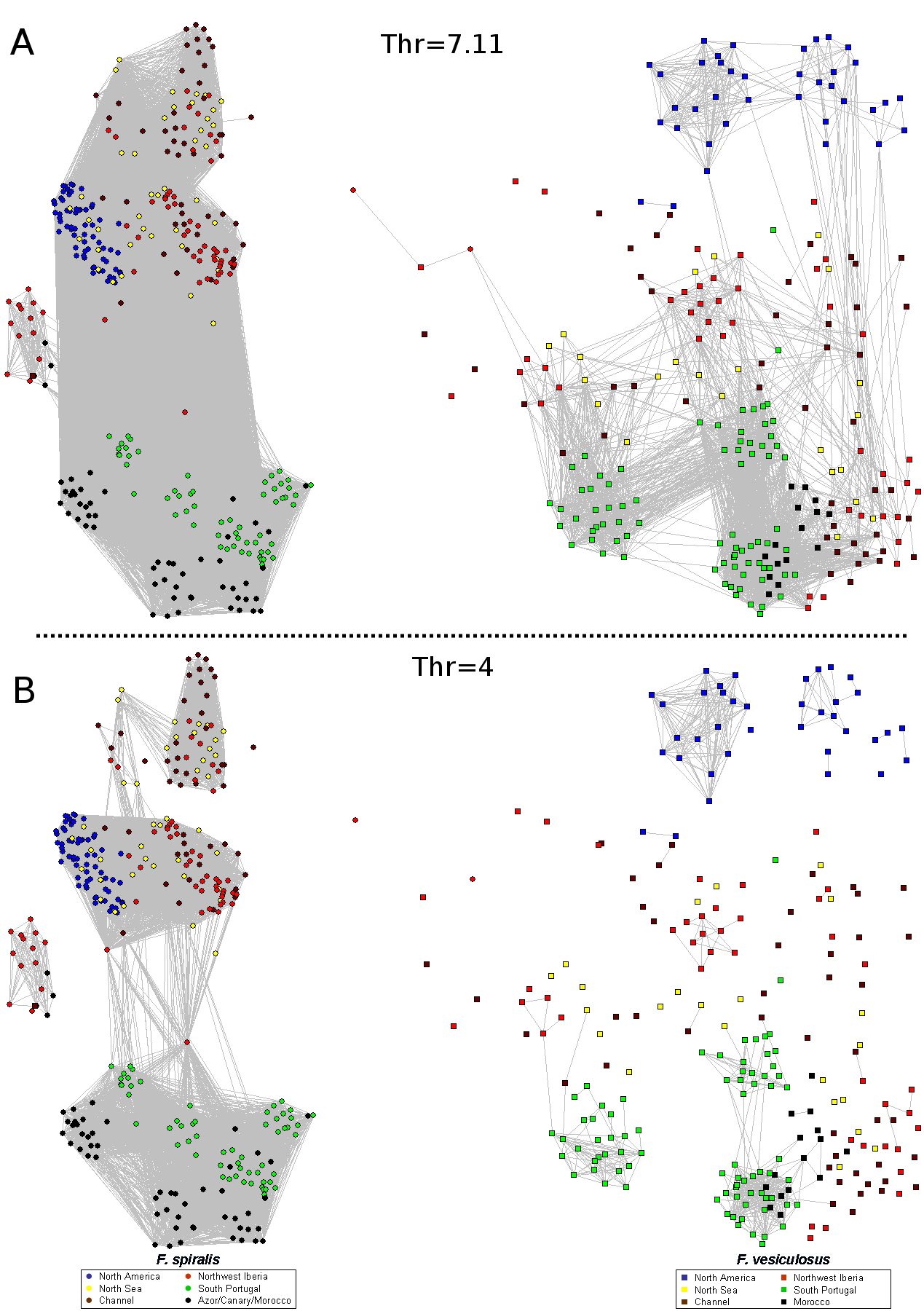

Supplement: Additional file 2 — Figure A2: Network topology of F. spiralis and F. vesiculosus individuals with the Rozenfeld distance under the percolation threshold. At the threshold values of 7.11 (A) and 4 (B). Nodes representing individuals are circles for F. spiralis and squares for F. vesiculosus. Colors correspond to geographical regions (see Figure A1). One can see at D = 7.11, the network is composed of two giant clusters corresponding respectively to the two species. At D = 4, the clusters of F. spiralis is still entirety at the exception of a little cluster of North Portugal. [file 1471-2148-11-33-S2.TIFF]

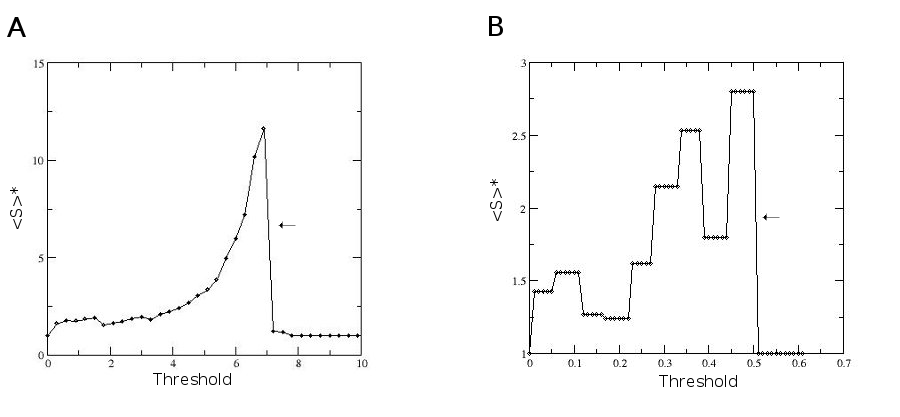

Supplement: Additional file 3 — Figure A3: The average cluster size excluding the largest one, as a function of the imposed genetic threshold obtained with the natural individuals. (A) calculated with the Rozenfeld distance, (B) calculated with the Shared alleles distance. The arrows indicate the percolation threshold (Dp) on the curves. [file 1471-2148-11-33-S3.TIFF]

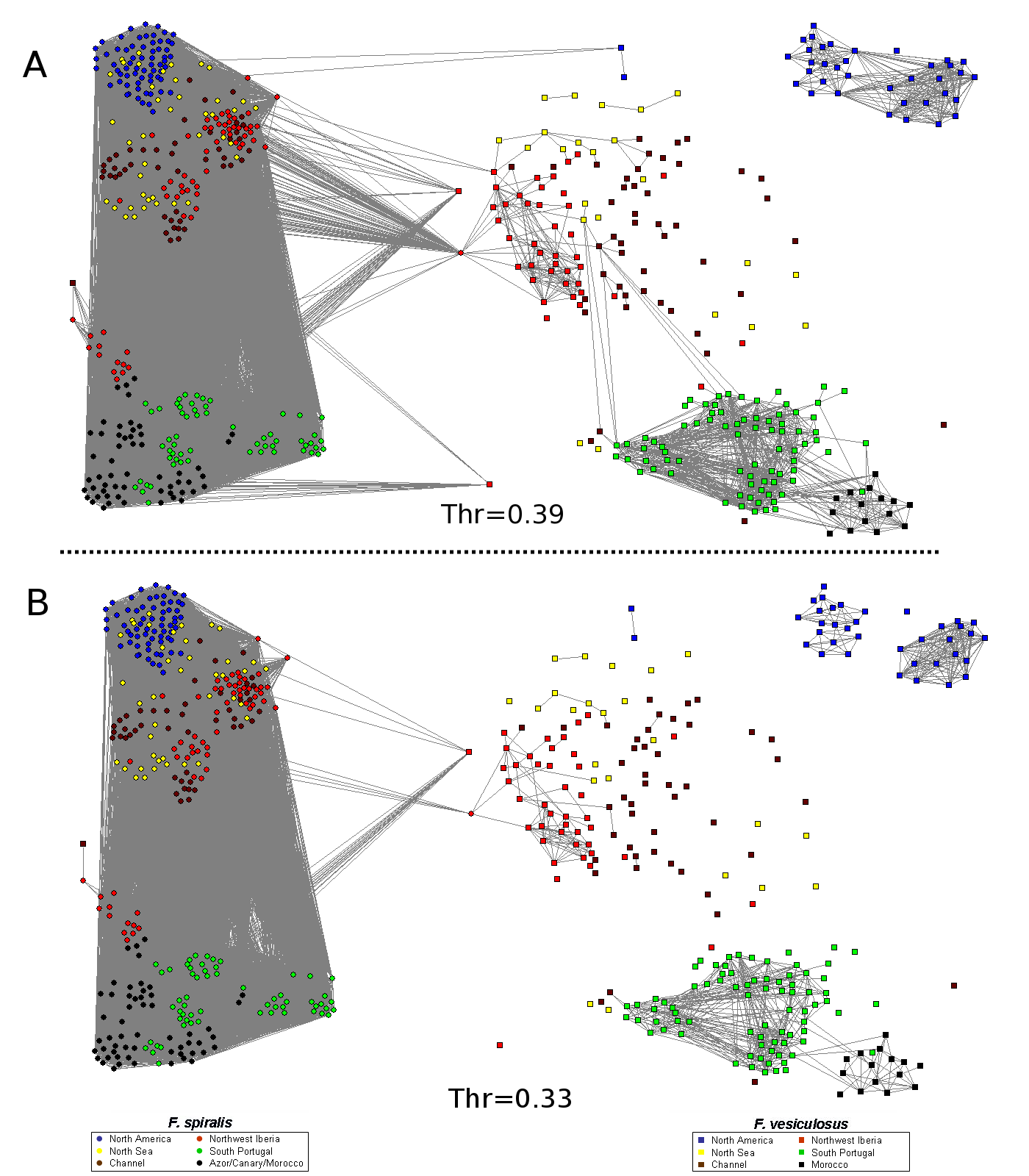

Supplement: Additional file 4 — Figure A4: Network topology of F. spiralis and F. vesiculosus individuals with the Shared alleles distance under the percolation threshold. At the threshold values of 0.39 (A) and 0.33 (B). Nodes representing individuals are circles for F. spiralis and squares for F. vesiculosus. Colors correspond to geographical regions (see Figure A1). [file 1471-2148-11-33-S4.TIFF]

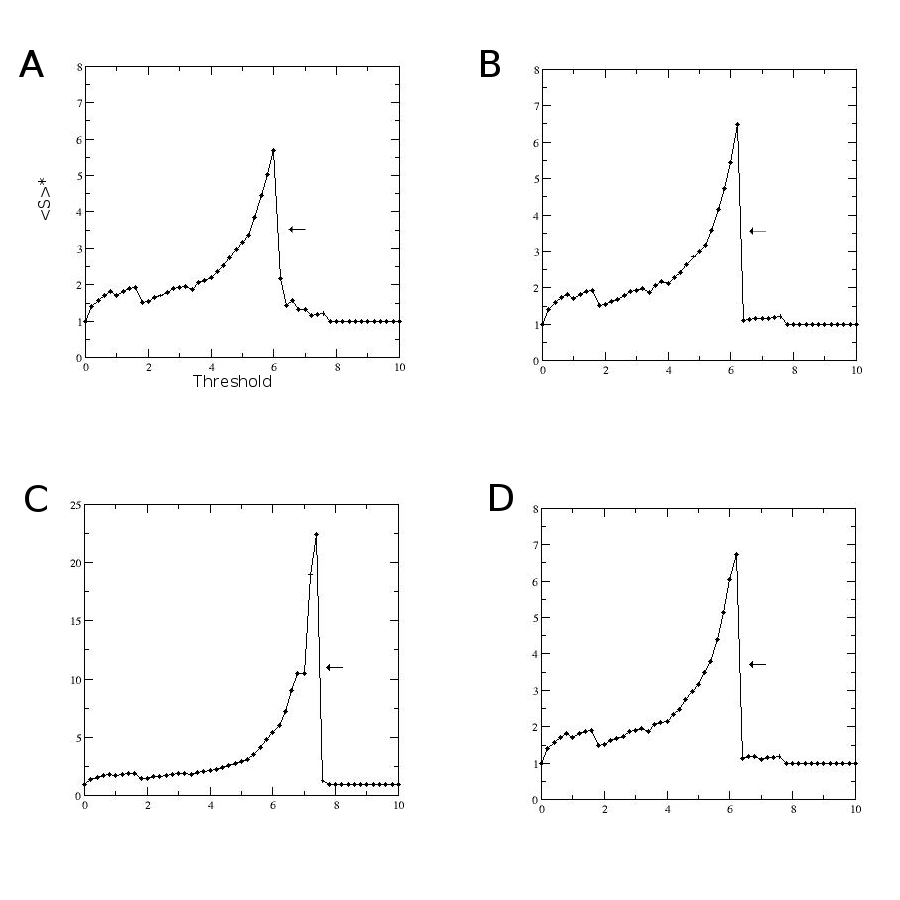

Supplement: Additional file 6 — Figure A5: The average cluster size excluding the largest one, as a function of the imposed genetic threshold calculated with the Rozenfeld distance. (A) is the curve obtained with the simulated hybrids spiralis/vesiculosus, (B) with back-crosses hybrids/spiralis, (C) back-crosses hybrids/vesiculosus and (D) with back-crosses hybrids/spiralis_vesiculosus. The arrows indicate the percolation threshold (Dp) on the curves. [file 1471-2148-11-33-S6.TIFF]

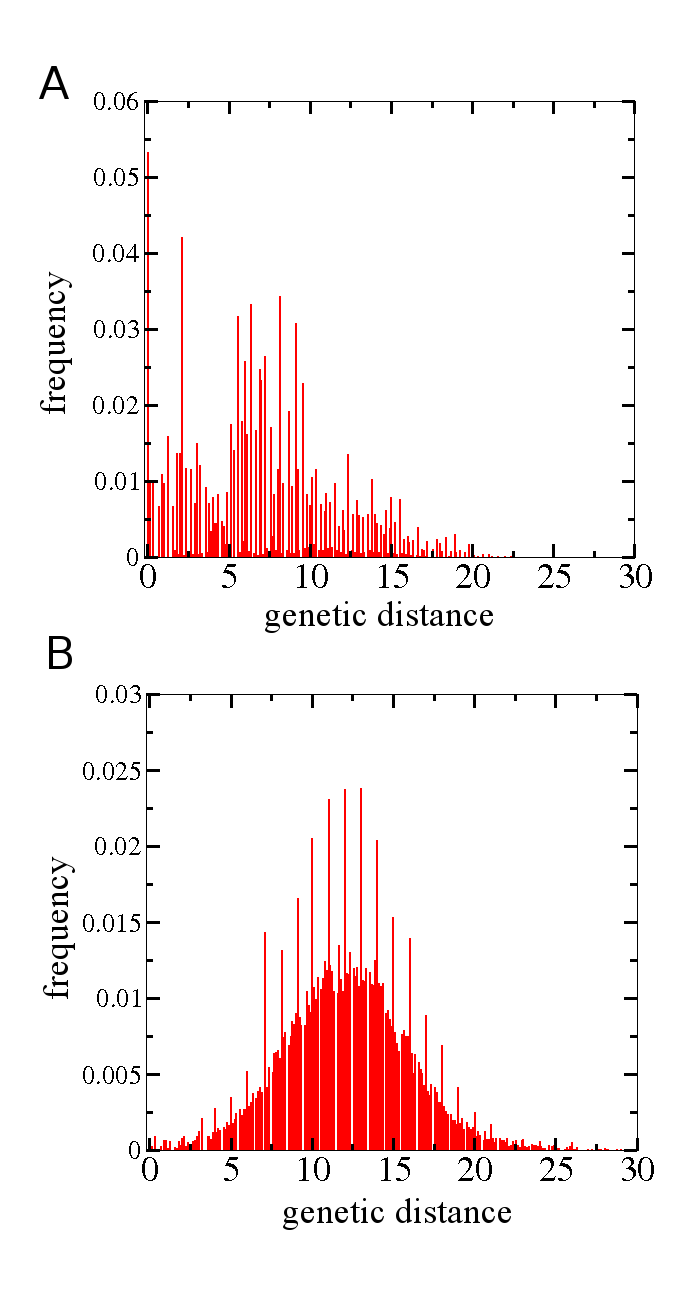

Supplement: Additional file 7 — Figure A6. The genetic diversity spectrum (GSD) for F. spiralis (A) and F. vesiculosus (B) based on the Rozenfeld distance. [file 1471-2148-11-33-S7.TIFF]

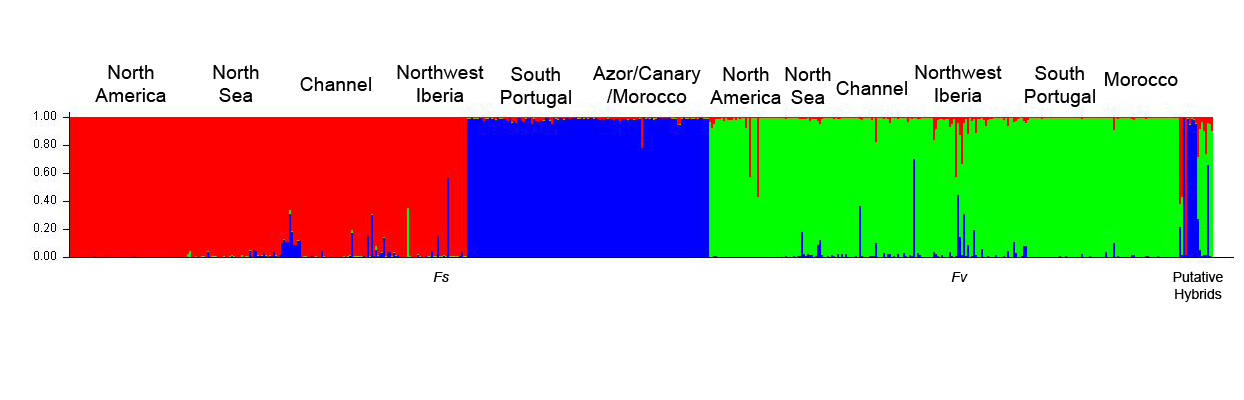

Supplement: Additional file 8 — Figure A7: Microsatellite detection of admixture (introgression) using the program STRUCTURE (Pritchard et al., 2000). Each individual is represented in the figure by a vertical bar and its colors indicate the proportional membership in each of k = 3 clusters, thereby providing a quantitative illustration of introgression. [file 1471-2148-11-33-S8.TIFF]
