# Supplementary material for: Travelling in time with networks: Revealing present day hybridization versus ancestral polymorphism between two species of brown algae, Fucus vesiculosus and F. spiralis
Source: BMC Evol Biol. 2011 Jan 31;11:33. doi: 10.1186/1471-2148-11-33 (PMC3040711; doi:10.1186/1471-2148-11-33)
Supplement: Additional file 5 — Hybridization simulation methods. This file includes the description of the method used to randomly generate individual hybrids. [file 1471-2148-11-33-S5.PDF]

**Additional File 5:**

**Hybridization simulation methods.**

A network is a holistic reconstruction in which overall and fine grained topologies are more or less depending on all the agents inserted. In order to avoid an over-representation of hybrid genotypes compared to the network built on original data, as these new “synthetic” individuals were included in the distance matrices, the natural putative hybrids identified on the basis of their position in the original SD network were therefore excluded and new networks were constructed including simulated hybrids.

Then, we have implemented a pipeline dedicated to the determination of number of links between simulated hybrids and real individuals of *F. spiralis* and *F. vesiculosus*. We have repeated 1000 times hybridization simulations and calculated each time the number of links present (see Table A3 below). Thus, we could use the links' distribution to select hybrid simulations data sets present around the median and used them to perform network analyses that get closer to the more probable situation of hybridization

As explained above, synthetic hybrids were built on the basis of the results obtained on the SD networks. For hybrids F1 the pool of parents was formed using the 88 individuals from the sympatric zone of Northwest Iberia that were not suspected of being hybrids (i.e. that exhibited no direct links with the individuals morphologically recognized as the other species at the threshold of 0.39), and for backcrosses F2 those same parents were crossed with one of the hybrids F1 generated before. In order not to disrupt the overall genotypic composition and consequent network topology, the generated number of simulated hybrids corresponded to the putative hybrids excluded as parents (17). The random variation in this synthetic hybridization process was estimated by repeating 1000 times 17 hybrids and illustrating the position of hybrids on the network by analyzing for each the links they shared with i) naturals individuals *F_spi*, ii) naturals individuals *F_ves* and iii) putative hybrids inside the matrix at the threshold of 0.39.

The results of simulations are described in Table A3 with the average, the standard deviation and the median values of links. On the basis of these results, datasets selected included hybrids

with the number of links close to the median values of simulations (see Table A4 below for the number of links obtained with the selected datasets)

Our Hybrids F1 exhibit numbers of links to natural *F_ves* and intra-hybrids similar to the “putative natural hybrids” but the number of links to natural *F_spi* is more than twice reduced (611 *vs* 1495). As expected, this number of links is 1.4 fold higher for the BC_*F_spi* (2043 *vs* 1495), while the number of links to natural *F_ves* is ten fold lower (3 vs 34). This tendency is reversed with BC_*F_ves*, only 68 links to naturals *F_spi* and 60 links to naturals *F_ves*. Finally, the closest values are reached by the hybrid BC_*F_spi_F_ves* with respectively 1043, 27 and 20 links to naturals *F_spi*, natural *F_ves* and intra-hybrids (versus 1495, 34 and 57).

**Table A3.** Hybrids link's distribution with natural *F_spi*, *F_ves* and between them after 1000 simulations.

| **Hyb_F1** | ***F_spi*** | ***F_ves*** | **intra_hybrids** |
|---|---|---|---|
| average | 633 | 30 | 60 |
| variance | 42097 | 28 | 59 |
| Standard deviation | 205 | 5 | 8 |
| median | ***626*** | ***30*** | ***59*** |
| **BC_*F_spi*** | ***F_spi*** | ***F_ves*** | **intra_hybrids** |
| average | 1579 | 5 | 68 |
| variance | 50523 | 6 | 16 |
| Standard deviation | 225 | 2 | 4 |
| median | ***1577*** | ***5*** | ***68*** |
| **BC_*F_ves*** | ***F_spi*** | ***F_ves*** | **intra_hybrids** |
| average | 95 | 75 | 28 |
| variance | 9689 | 132 | 39 |
| Écart-type | 98 | 11 | 6 |
| median | ***57*** | ***74*** | ***27*** |
| **BC_*F_spi_F_ves*** | ***F_spi*** | ***F_ves*** | **intra_hybrids** |
| average | 1158 | 36 | 28 |
| variance | 36154 | 64 | 31 |
| Standard deviation | 190 | 8 | 6 |
| median | ***1149*** | ***36*** | ***28*** |

**Table A4.** Links' number between simulated hybrids datasets selected and natural *F_spi*, *F_ves* and between them at the threshold of 0.39 (SD network).

| | *F_spi* | *F_ves* | intra hybrids |
|---|---|---|---|
| natural data | 1495 | 34 | 57 |
| hybrids F1 | 611 | 30 | 74 |
| Back-crosses *F_spi* | 2054 | 3 | 63 |
| Back-crosses *F_ves* | 68 | 60 | 20 |
| Back-crosses *F_spi_F_ves* | 1042 | 27 | 20 |
